# Bridging digital health gaps in South Africa: A qualitative study of the digital divide, interoperability and health equity

**DOI:** 10.1177/20552076261440967

**Published:** 2026-04-17

**Authors:** Trust Saidi, Pavel Nenad, Parisa Gazerani, Minna Annika Pikkarainen

**Affiliations:** 1 Department of Product Design, Faculty of Technology, Art and Design, 60499 Oslo Metropolitan University, Oslo, Norway; 2 Department of Life Sciences and Health, Faculty of Health Sciences, 60499 Oslo Metropolitan University, Oslo, Norway; 3 Department of Rehabilitation and Health Technology, Faculty of Health Sciences, 60499 Oslo Metropolitan University, Oslo, Norway

**Keywords:** digital divide, interoperability, digital health equity, health information systems, institutional capacity, South Africa

## Abstract

**Objective:**

This study investigates how the interplay between the digital divide and limited interoperability in healthcare technologies contributes to regional disparities in healthcare access and outcomes in South Africa. It further examines how digital health systems might be leveraged to reduce inequities and promote more inclusive development.

**Methods:**

The research draws on qualitative interviews with clinicians, digital health experts, policymakers, and implementers across public and private sectors. Data were analysed thematically, with attention to structural conditions shaping digital readiness, institutional capacity, information flow, and user experience across provinces.

**Results:**

Findings suggest that structural barriers, such as uneven broadband access, outdated infrastructure, staff shortages and institutional fragmentation, interact with weak interoperability to produce inconsistent digital health experiences. These constraints limit continuity of care, restrict patient mobility across systems and contribute to the existing disparities between better-resourced and under-resourced regions. Participants emphasised that digital tools alone cannot deliver equitable outcomes without coordinated governance, national digital public infrastructure, and sustained organisational readiness.

**Conclusion:**

Digital transformation will only advance equity when digital access, interoperability and institutional capacity are addressed as interdependent system-level requirements. Strengthening national digital infrastructure and governance is essential to ensuring that digital health technologies narrow, rather than reproduce, existing inequalities, including persistent regional disparities that manifest within the broader national system. Sustained national investment in digital governance and shared infrastructure is indispensable to ensuring that digital health systems mitigate rather than reinforce deep-seated inequities. Embedding equity in the design and implementation of digital health initiatives is essential for translating technological progress into inclusive health outcomes.

## Introduction

Digital technologies have become integral to contemporary health systems, driving expectations that they can enhance access, efficiency, and continuity of care. However, their rapid expansion has intensified concerns that digital transformation may reproduce or deepen existing health inequities rather than alleviate them.^1–3^ These tensions converge around three interrelated domains widely recognised as central to equitable digital health: the digital divide, interoperability, and health equity. Although each has been studied extensively in isolation,^4,5^ what remains less examined is how these strands of work can be brought together empirically to understand their combined influence on equitable outcomes, particularly within health systems marked by structural inequalities and fragmented digital infrastructures.^6,7^

Research on the digital divide, interoperability, and health equity has generally proceeded along parallel trajectories, resulting in limited synthesis of how these domains intersect to influence equitable health outcomes. Interoperability scholarship has tended to focus on technical and semantic challenges in achieving seamless data exchange,^8,9^ while digital divide studies foreground disparities in access, affordability, digital literacy, and meaningful engagement.^10^ Health equity frameworks increasingly incorporate digital determinants of health but rarely embed them in analyses of fragmented digital ecosystems. A fundamental misalignment is evident: digital health uptake continues to accelerate, yet the infrastructural and governance foundations needed for equitable interoperability lag significantly behind.^11,12^ This fragmentation is consequential: when digital exclusion intersects with non-interoperable health information systems, inequities can be magnified, limiting the reach, utility, and fairness of digital health initiatives.^13,14^ Understanding these interdependencies requires empirical evidence that moves beyond high-level conceptual frameworks in order to address the missing links toward more equitable digital health systems.^15,16^

Although each component of the digital divide, interoperability, and health equity has been widely studied, their interdependence and its impact on practice have received limited scholarly attention. As a result, context-specific implementation strategies targeting this nexus are still notably lacking.^17^ This fragmentation in the literature mirrors broader disconnection within the system itself, as the very factors that most profoundly influence whether digital health can advance equity or not are often studied in isolation. South Africa presents a contrasting case of technologically advanced infrastructure co-existing with persistent health inequities.^18^ The country offers a particularly compelling context for interrogating these dynamics, given its markedly fragmented health system in which public and private sectors operate across numerous non-interoperable digital platforms.^19^ Despite ambitious national strategies aimed at strengthening digital health capabilities,^20^ longstanding socioeconomic and spatial inequalities, pronounced rural–urban digital divides, and the structurally fragmented health system create highly uneven conditions for digital participation and system integration.^21^ In addition, the hospitals in the country adhere to heterogeneous and frequently incompatible standards, which further undermines system-wide data interoperability.^19^ While targeted digital initiatives have emerged, broader challenges persist, including affordability barriers, infrastructural constraints, and inconsistent standards for digital tool deployment.^22^

This paper addresses a key gap in the digital health equity literature by examining how structural barriers in digital access and limitations in system interoperability jointly shape the distribution of healthcare opportunities beyond the immediate provincial settings in South Africa, especially among the marginalised communities. South Africa was selected as a unit of analysis of this study because its stark geographic and infrastructural variation - from urban provinces with advanced digital capability to rural regions facing persistent connectivity and resourcing constraints - provides a unique setting in which to examine how the digital divide and interoperability gaps interact. The marginalised groups, such as rural communities with limited infrastructure, low-income households, and individuals dependent on under-resourced public healthcare facilities are the most affected by digital exclusion in South Africa. Together, these dynamics shape who can access digital pathways, how their clinical information flows, and whether digital health initiatives translate into meaningful improvements in care. This intersectional lens provides a more nuanced understanding of the systemic conditions required for equitable digital transformation in contexts characterised by infrastructural unevenness, institutional fragmentation, and longstanding patterns of exclusion. Accordingly, the central research question guiding this study is: How does the interplay between the digital divide and lack of interoperability in healthcare technologies contribute to regional disparities in health equity outcomes in South Africa, and how can these technologies be harnessed to promote equitable development?

### The digital divide, interoperability and equity as interlinked and co-producing constructs

This framework treats the digital divide, interoperability and equity as interdependent constructs that co-produce digital health outcomes. The digital divide is conceptualised as a multidimensional and hierarchical phenomenon comprising three interconnected levels. First-level divides encompass disparities in physical access to devices, connectivity, and service coverage; second-level divides concern differences in digital skills, literacies, confidence, and effective usage; and third-level divides refer to inequalities in the ability to convert digital engagement into meaningful benefits and outcomes.^23,24,25^ Recent empirical and theoretical work consistently supports this three-tiered understanding.^10,26^ The digital inequities increasingly stem not only from access gaps, but also from skill-based and outcome-based differentials that shape who meaningfully benefits from digital transformation. These divides reflect and reinforce broader structural conditions such as socioeconomic position, geography, disability and institutional arrangements, meaning that simply providing technology seldom guarantees meaningful participation.^27^

Interoperability refers to the capacity of heterogeneous health information systems to communicate, exchange, and meaningfully use data across organisational and technological boundaries.^8^ Using standardised coding systems enhances system interoperability by enabling consistent data interpretation and smooth information exchange, thereby supporting more coordinated and integrated healthcare delivery.^8,28^ It is widely recognised as a critical requirement for digital transformation, efficient data use, and integrated service provision.^29^ Health equity denotes fair and just opportunities for all to attain optimal health; it goes beyond equal allocation by addressing structural barriers and distributional injustice. Effective pursuit of health equity requires integrated approaches that consider social determinants, power structures, intersectionality, and historical context, alongside ongoing measurement and adaptive interventions.^30^

The digital divide serves as a foundational access gate, determining who enters the digital health ecosystem and under what conditions. Access to digital health extends beyond mere connectivity to include affordability, device reliability, digital literacy, usability, and trust/privacy expectations that influence how organisations adopt, integrate, and operationalise digital health system.^31,32^ After entry, interoperability governs what happens inside the system by determining whether interactions are coherent, safe, efficient, and continuous across settings and time. Even when technical access barriers are reduced, limited interoperability across digital health systems can still produce fragmented or siloed records that hinder information flow and create duplicated tasks and administrative burdens - issues widely observed in healthcare settings where inconsistent data formats, disconnected platforms, and poor integration slow decision-making and reduce efficiency.^33,34^ In this sense, interoperability mediates the translation of “access” into “benefit”: when systems cannot exchange and reuse data in ways that facilitate workflow alignment, the returns to digital access are diminished and unevenly distributed.^35^ Conversely, when technical standards, semantic mappings, governance arrangements, and organisational processes align, digital health more reliably delivers continuity and reduces error propagation across the care journey.^8^

In this conceptualisation, equity arises from the interaction of access and interoperability, which implies that aligning meaningful access with robust connectivity improves availability, accessibility, acceptability, and quality.^32^ This includes ensuring consistent service reach, reducing barriers such as cost, literacy, and interface complexity, supporting cultural and linguistic relevance as well as privacy expectations, and maintaining high standards of safety, continuity and error reduction.^36^ Positioning equity inside the causal logic clarifies that interventions should be designed with both upstream access conditions and downstream system functioning in view. In this framework, equity emerges not as an abstract norm but as the observable distribution of burdens such as repeated registrations, delayed diagnostics, or fragmented care across differently resourced populations and settings. The framework specifies a sequential causal chain moderated by cross-cutting factors, including affordability and economic shocks, digital literacy and user competencies, language and cultural acceptability, privacy and data-governance mechanisms, device and infrastructure reliability, standards conformance and record-linkage practices, institutional coordination across providers, and routine data quality for monitoring and feedback.^14,37,38^ These factors act as levers that amplify or dampen inequitable effects at each stage: for example, improvements in access without adequate system connectivity often stall before producing equitable outcomes, while strong interoperability without attention to affordability or literacy may still exclude those least advantaged.^32^ Taken together, this framework conceptualises digital health equity as emerging from the interaction between digital access conditions and system interoperability, providing the analytical basis for examining how these dynamics shape regional disparities in healthcare delivery.

### Research methodology

This study investigated digital health in South Africa through a qualitative lens, employing a constructivist paradigm to examine the lived experiences and perspectives of professionals involved in digital health implementation. The study adopted the constructivist approach,^39^ which prioritises the co-construction of meaning within specific social, institutional, and technological contexts. It suits this study because it enables a deeper understanding of how digital health systems are interpreted and shaped by those who implement them, emphasizing context over abstraction. The study design was informed by the need to understand complex socio-technical dynamics in healthcare settings, which are best captured through in-depth, context-sensitive qualitative inquiry.

A total of 13 participants were recruited using purposive and snowball sampling strategies, and they were recruited via email, including 10 digital health experts and 3 medical doctors, all with relevant experience in South Africa. For this study, “digital health experts” were defined as individuals with (a) a minimum of five years of demonstrable experience in designing, implementing, or managing digital health systems; (b) professional roles directly related to digital health strategy, governance, or system integration; and (c) current or recent involvement in provincial or organisational digital health initiatives. These criteria ensured that expert status was based on professional practice rather than job titles alone. While the study draws on experts based in two provinces, many participants held multi-provincial or national responsibilities that provided insight into system-wide digital health dynamics. Accordingly, the national framing used in this paper reflects analytic patterns derived from their broader professional exposure, rather than claims of geographic representativeness, and the findings were interpreted with this scope in mind.

The sample size for this study (n=13) was based on the information power framework, whereby the sufficiency of the sample is assessed in relation to the specificity of the study aim, the quality of dialogue, and the richness of the data generated.^40^ Participant heterogeneity was intentionally built into the sampling strategy to capture variation across roles, sectors, and experience levels. Participants included digital health consultants, information technology managers in provincial health departments, system architects, data governance specialists, and clinicians using electronic health records or telemedicine platforms. Professional experience ranged from 5 to 20 years. Sector representation included public and private health organisations. A detailed description of the participants is presented in Table 1 to illustrate heterogeneity across roles, experience, and institutional affiliations.

**Table 1.** Characteristics of participants selected for the study based on their involvement in multiple provincial and/or national digital health roles.

Characteristic	Category	Number
Role group	Medical doctors/clinicians (EHR/telemedicine users)	3
	Digital health experts	10
	*-Digital health consultants*	*3*
	*-IT managers (provincial health department)*	*3*
	*-System architects/Solutions engineers*	*2*
	*-Data governance/HIE specialists*	*2*
Sector	Public	7
	Private/NGO	6
Years in digital health	5–9 years	3
	10–14 years	4
	15–20 years	6
Primary digital domain	Telemedicine/virtual care	4
	Interoperability/HIE integration	5
	Data governance/standards	4
Total participants	​	13

The interview guide was developed based on a conceptual analysis of the digital divide and interoperability frameworks (see Interview Guide in Appendix 1). The guide was pilot tested with two experts to ensure clarity and relevance, and minor adjustments were made accordingly. Semi-structured interviews were conducted online via secure video conferencing platforms between March and May 2025. Each interview lasted between 45 and 60 minutes. All interviews were conducted in English, audio-recorded, and transcribed verbatim. All respondents provided informed consent to be recorded and were clearly informed about how the data would be used. All recordings and transcripts were securely stored on encrypted institutional servers, accessible only to the research team. The interviews were transcribed and anonymised, with no real names or personal information retained from participants, to ensure confidentiality and adhere to ethical research standards. Ethical approval was granted by the Norwegian Agency for Shared Services in Education and Research to ensure compliance with privacy protection regulations.

Data analysis followed Braun and Clarke’s^41^ a six-phase framework for thematic analysis, which includes familiarisation with the data, generating initial codes, searching for themes, reviewing themes, defining and naming themes, and producing the report. Initially, each researcher independently performed thematic coding in three interviews, following general principles of open coding in qualitative research.^42,43^ Initial coding followed the principles of reflexive thematic analysis, with researchers generating inductive, flexible codes that captured patterned meaning across the dataset. The thematic codes were largely consistent across researchers, demonstrating shared interpretive understanding through joint coding. Subsequently, a preliminary coding framework was established, which the first researcher applied to the remaining interviews. Building on this, the codebook was refined to reflect the paper’s conceptual chain and the empirical salience of organisational/institutional dynamics.

The initial codes moved beyond generic categories to target organisational/institutional features of the digital divide (e.g., sectoral resourcing, provincial autonomy, facility-level infrastructure/power/hardware,), post-entry interoperability mechanisms (e.g., intra-facility fragmentation across departmental systems; cross-facility/provincial non-alignment; private–private data silos), and equity implications as distributional outcomes (i.e., how benefits/burdens are allocated across settings and populations, rather than individual-level barriers). They were consolidated into three higher-order themes aligned to the framework: (1) the organisational/institutional digital divide as the ‘entry gate’; (2) interoperability as the system mechanism that converts access into burden or benefit ‘after entry’; and (3) equity as the evaluative outcome, expressed as empirically observed patterns of who gains coordinated, continuous care versus who absorbs duplication, delay, and risk. A fourth cross-cutting theme captured actionable institutional levers that mitigate the above mechanisms. In line with reflexive thematic analysis, theme development was an interpretive, researcher-active process rather than a procedural coding hierarchy. Throughout the analysis, we maintained an audit trail documenting coding decisions, theme refinement, and researcher reflections. NVivo software was used to manage and organise the data. We conducted member checking with three participants to enhance the credibility of the findings; participants confirmed that the interpretations accurately reflected their experiences, and minor refinements were made based on their feedback.

## Results

The results from the study present evidence on how the combined effects of the digital divide and interoperability gaps in healthcare technologies shape regional patterns of healthcare access and outcomes in South Africa.

## The entry gate to digital health

Participants indicated that access to digital health in South Africa is heavily determined by organisational and institutional factors, including sectoral resourcing, facility-level infrastructure and governance structures. These factors play a decisive role in determining which organisations can adopt and sustain digital health technologies and which cannot. It emerged from data that digital health capabilities are concentrated in well-resourced private facilities, while public healthcare facilities, which serve the majority of South Africans, face significant barriers to adoption. Participants consistently perceived the inequities in sectoral resourcing:

Digital health is exclusively a private healthcare offering… There are no digital healthcare offerings in the public sector that I know of, at any large scale.

Public sector challenges, including constrained budgets, staff shortages, and limited operational support, were identified as structural impediments to digital health adoption. One interviewee noted the systemic resource limitations:

Doctors were actually not getting employed by the state because the state didn’t have enough resources to actually employ doctors… this goes far beyond healthcare.

The analysis suggests that South Africa’s provincial autonomy exacerbates fragmentation in governance and procurement, creating divergent organisational systems that hinder scalability and connectivity. According to a digital health expert:

In South Africa, provinces have their own autonomy in how they do things… This autonomy institutionalises silos as a default rather than an exception.

This decentralisation was found to impede the creation of a unified national digital health infrastructure, further complicating cross-province healthcare delivery. A participant underscored how this affects planning and coordination:

When you have this decentralised model, coming up with a more contextual plan for the entire country becomes more difficult, because there will be no coordination and cooperation between these different entities for accurate and timely reporting.

It was noted that at the facility level, outdated hardware, unreliable connectivity, and power disruptions especially due to load shedding were identified as significant barriers to digital health adoption. The physical and technological infrastructure in many public facilities is inadequate to support even basic digital systems, as described by one medical doctor:

The computer hardware infrastructure is quite old, it doesn't get replaced, runs out of memory, and becomes very, very slow and cumbersome to use.

Even in relatively advanced provinces such as the Western Cape, load shedding disrupts internet access and undermines the reliability of digital health services, further eroding trust in these systems.

Bureaucratic inefficiencies, including complex procurement processes and delays in policy implementation, further inhibit digital health adoption. One participant highlighted the systemic delays caused by red tape:

South Africa is notorious for its red tape; everything is legislated… So, things take long to get approved.

Moreover, while policies exist, they are often not implemented effectively, as one clinician explained:

The policies are there, but they are not being implemented… Six years later, it is not implemented because there is no drive to implement it.

This observation indicates a recurring implementation gap in the health system, where formal policy commitments are not matched by operational follow-through, enforcement mechanisms, or sustained leadership attention.

### Post-entry interoperability mechanisms and equity implications

The results showed that, for facilities that manage to overcome the barriers to entry, the lack of interoperability in digital health systems determines whether access leads to coherent and continuous care or to inefficiencies, duplication, and delays.

Within hospitals, digital systems are often siloed by department, requiring clinicians to navigate multiple standalone systems. This lack of integration undermines seamless care and forces staff to rely on inefficient workarounds. One digital health expert described the issue:

If I want to look at the lab results, I need to go into a different system. If I want to look at the X-rays, I need to go into a different system. Nothing is coordinated into one single system.

Such fragmentation disrupts referral pathways, as noted by a medical doctor:

“If I refer a patient to a different department within the hospital, I don't know what system they're using… everyone is doing their own thing. I end up making a phone call instead.”

These inefficiencies adversely impact under-resourced facilities, where additional administrative burdens are harder to absorb, further lowering the quality of care.

It was found that at the provincial levels, the lack of aligned digital systems and shared patient identifiers disrupts the continuity of care. Patients who move between facilities or provinces for referrals, employment, or other reasons often face disconnected systems that fail to communicate with one another. This results in repeated registrations, duplicate testing, and delays in care delivery. One participant explained the consequences of these gaps:

Without a national system, digital health does not accommodate cross-border transactions. For example, a lot of people from the Eastern Cape come to the Western Cape for medical services, but the digital systems do not communicate with each other.

These gaps disproportionately burden mobile and underserved populations, particularly those reliant on public services, compounding existing inequities.

It was shown in the study that the private healthcare sector, despite its robust resources, remains fragmented due to competition between private providers. This results in silos that limit the sharing of information, even within the private sector. A medical doctor perceived this challenge:

Despite the fact that the private sector is well resourced, the challenge is that private hospitals are run by different companies, which adversely affects the integration of digital health. And obviously, they are competing with each other.

For patients who move between private and public healthcare systems, the lack of interoperability creates administrative friction, necessitating re-tests and re-triaging when transitioning into public facilities. Public providers should absorb these spillover burdens, further straining their limited resources.

Participants indicated that even when digital systems are deployed, the operational reliability of these systems is affected by aging hardware, power outages, and poor connectivity which determines whether they remain usable. Load shedding, in particular, was identified as a recurring issue that undermines the reliability of digital health platforms, even in relatively developed provinces. As one expert noted:

Connectivity issues persist, particularly exacerbated by load shedding, which disrupts internet access and hinders the reliability of digital health services.

Furthermore, it was found in the study that successful implementation of digital systems depends on institutional readiness, including adequate training, workflow redesign, and change management processes. Without these, adoption often fails, as clinicians and staff become overburdened or disillusioned. One expert explained:

Clinicians and nurses and allied health care workers need to be conditioned. You can't just drop a system and expect it to work… this is a transformational journey.

These reflections underscore that digital transformation is not simply a technical rollout but a profound organisational shift that needs time, leadership commitment, and sustained capacity building. Participants indicated that when systems are introduced without aligning them to clinical routines or without sufficient preparation, frontline workers experience them as additional burdens rather than tools that enhance care.

### Actionable institutional levers to mitigate digital health inequalities

Participants repeatedly emphasised that infrastructural and institutional barriers including outdated and unreliable hardware, intermittent or weak connectivity, frequent load-shedding related power disruptions, and slow or inconsistent procurement processes disproportionately affected facilities serving low-income and rural populations. These constraints limited digital capacity and produced more frequent system downtime, greater reliance on manual workarounds, and delayed access to clinical information. For example, the shared infrastructure, referred to as “digital highways,” would provide the foundation for integrating disparate systems and ensuring secure, seamless data exchange. One participant used an analogy to describe the role of government in this process:

The analogy I would use is that we rely on the government to create highways for us, which we want to drive on our own cars. And we need to do something similar for digital health… unique identifiers… a common infrastructure that manages identity, privacy, public key infrastructure.

Participants also emphasized the importance of coordinated governance to ensure successful implementation of such infrastructure. Reflecting on the lessons learned from COVID-19, one interviewee noted:

We can build an integrated system, we can build it quite quickly. And the way to do that is to do it in a highly collaborative way, well-coordinated by a central facilitator who facilitates us in good faith around a common goal. Immediately after COVID, we seem to have now poor facilitation, poor coordination, poor collaboration.

Participants further stressed that such national infrastructure is not optional but foundational. As one interviewee explained:

For a national health insurance… there needs to be a digital backbone… aggregation of data… digital backbone is a prerequisite).

This reinforced the argument that without shared national rails, large-scale reforms such as National Health Insurance cannot function effectively.

The success of coordinated digital health efforts during the COVID-19 pandemic provides a valuable model for future implementation. Lessons from this period indicate that centralised facilitation and collaboration across stakeholders can accelerate progress toward shared goals. These efforts should be institutionalised to ensure sustained governance and collective action in advancing digital health equity as indicated below:

We need a common infrastructure that manages identity, privacy, and public key infrastructure… It’s the job of the government to deliver it and invite providers to use it.

It was revealed in the study that organisational readiness also depends on change management the training, workflow redesign, and time protection required for staff to use systems consistently at scale. Participants stressed that implementation is a transformational journey, that needs careful planning to make the process manageable:

“You also don't want them to be overburdened… then they will stop using the system.

The participants reported that when system-related demands feel excessive, users reduce or stop their use of the system. The process of maintaining manageable workload demands was identified as a key condition for continued use.

## Discussion

The findings show that the digital divide and interoperability are deeply interconnected, working together to shape inequities in healthcare delivery and outcomes. This discussion explores the implications of these findings, situating them within the broader conceptual framework while offering pathways to harness digital health technologies for equitable development.

The digital divide in South Africa operates at multiple levels, creating a foundational barrier to equitable access to healthcare. As conceptualised in the framework, the digital divide encompasses disparities in physical access, digital skills, and the ability to derive meaningful benefits from technology.^26,27^ The study findings align with this understanding, demonstrating how access to digital health systems is stratified by organisational resourcing, geographic location, and systemic inequalities. Public healthcare facilities serving the majority of the population struggle to adopt and sustain digital health systems due to limited infrastructure, unreliable hardware, and frequent power outages caused by load shedding. These barriers exemplify the first-level divide, where basic access to devices and connectivity is unevenly distributed.^44^ At the same time, limited digital literacy and inadequate training among healthcare workers further constrain the ability of public sector organisations to engage effectively with digital health technologies. This echoes the second-level divide, which highlights the importance of skills and confidence in determining effective usage and system uptake.^26^ Importantly, even when access barriers are overcome, the third-level divide, which manifests in the form of inequities in converting digital engagement into meaningful outcomes.^45,46^ Public facilities often experience operational inefficiencies, fragmented uptake, and diminished returns on their digital investments. These findings support the notion that digital inequities are not solely a function of access but are shaped by broader structural conditions, including socioeconomic disparities, geographic isolation, and limited institutional capacity.^32^

The study has revealed that interoperability is critical for enabling heterogeneous health information systems to exchange and meaningfully use data across organisational boundaries. The study found significant interoperability gaps across South Africa’s healthcare ecosystem, resulting in fragmented care and inefficiencies. At the intra-facility level, siloed departmental systems require clinicians to navigate multiple platforms, undermining seamless care and increasing administrative burdens. At the inter-facility and inter-provincial levels, non-aligned systems and the absence of shared patient identifiers disrupt the continuity of care for patients who move between regions or providers. These findings are consistent with previous research, which highlights how inconsistent data formats, disconnected platforms, and poor integration create barriers to efficient information flow.^33,34^ The study also highlights the persistence of institutional silos within the private healthcare sector, where competition and legal constraints limit data sharing between providers. This further reinforces inequities, as private sector resources remain inaccessible to the broader healthcare system, while public facilities bear the spillover burden of incomplete records and duplicated efforts. These challenges illustrate how interoperability mediates the translation of access into benefit, determining whether digital health technologies produce continuity and improved outcomes or exacerbate inefficiencies and inequities.^35^

Equity, as defined in the framework, represents the cumulative distribution of benefits and burdens across populations, emerging from the interaction between access and interoperability.^32^ The findings suggest that South Africa’s digital health ecosystem is far from equitable, as benefits are concentrated in well-resourced private facilities, while under-resourced public facilities experience the greatest burdens. This inequity reinforces historical and structural disadvantages, excessively affecting rural populations, mobile patients, and those with lower socioeconomic status. For example, fragmented systems and interoperability gaps lead to duplicated testing, delayed results, and administrative inefficiencies, which are harder to absorb in under-resourced settings. These findings align with prior research showing that fragmented digital ecosystems erode the availability, accessibility, acceptability, and quality of care, particularly for marginalised populations.^36^ Conversely, where interoperability is achieved through shared infrastructure, governance, and standards, digital health systems can deliver continuity, reduce administrative burdens, and support equitable outcomes.^8^ This underscores the critical role of interoperability in fostering health equity by ensuring that digital health systems function as cohesive, integrated networks rather than fragmented silos.

The findings highlight several pathways, through which digital health technologies can be leveraged to reduce inequities and promote equitable development. These strategies emphasises the need for integrated, multi-level approaches that address both upstream access conditions and downstream system functioning.^32^ Addressing the first-level digital divide involves targeted investments in infrastructure, including reliable connectivity, modern hardware, and power stability. These investments are particularly critical in under-resourced public healthcare facilities, where infrastructure gaps are most pronounced. Without addressing these foundational barriers, digital health initiatives risk deepening existing inequities rather than reducing them. Digital health systems cannot succeed without the requisite human capital and institutional capacity to support their implementation and use. Addressing the second-level digital divide requires investments in training, capacity building, and change management to ensure healthcare workers can effectively engage with digital technologies. Digital literacy and user competencies are key levers for amplifying the benefits of digital health.^26^

The findings underscore the need for a unified national digital infrastructure to address the fragmentation created by interoperability gaps. This includes implementing shared health information exchanges, unique patient identifiers, and standardised data formats to enable seamless communication between systems. These shared “digital highways” are critical for enabling the secure and efficient exchange of health data across organisations, provinces, and sectors.^8^ Without this foundational infrastructure, efforts to achieve equitable digital health outcomes will remain fragmented and piecemeal. Furthermore, a centralised governance framework is essential for aligning policies, standards, and practices across South Africa’s decentralised healthcare system. The findings suggest that provincial autonomy often creates silos that hinder collaboration and scalability. A unified governance structure could facilitate coordinated planning, consistent policy implementation, and system-wide interoperability, ensuring that digital health technologies serve as tools for equity rather than division.^35^ To ensure that digital health technologies promote equity, it is imperative that interventions should prioritise the needs of the most marginalised populations.). Equity-focused interventions should also incorporate mechanisms for continuous monitoring and evaluation, enabling adaptive responses to emerging disparities.^14^

## Recommendations informed by the findings

Policymakers need to prioritise equity in digital health strategies, ensuring that resource allocation, infrastructure development, and regulatory frameworks explicitly address the needs of underserved regions and populations. This approach should include establishing universal standards for system interoperability and creating a national health information exchange to bridge the public-private divide.

The success of coordinated digital health efforts during the COVID-19 pandemic provides a valuable model for future implementation. Lessons from this period suggest that centralised facilitation and collaboration across stakeholders can accelerate progress toward shared goals. These efforts should be institutionalised to ensure sustained governance and collective action in advancing digital health equity.

Future interventions should incorporate measurable equity indicators into their monitoring and evaluation frameworks, focusing on aspects, such as access, usability, continuity of care, and outcomes for marginalised populations. Regular feedback loops, supported by high-quality data, can help identify gaps and drive adaptive improvements in digital health systems.

Continued research is needed to explore the nuanced ways in which the digital divide and interoperability gaps intersect with social determinants of health, including gender, disability, and geographic disparities. Sharing best practices and lessons learned from successful initiatives both within South Africa and globally can inform more effective, and contextually relevant solutions.

Finally, it is crucial for South Africa to work toward a digital health ecosystem that is inclusive by design. This includes addressing cultural and linguistic barriers, ensuring that technologies are accessible to users with varying levels of digital literacy, and engaging communities in the design and implementation process. By prioritising the voices and experiences of the most marginalised, digital health initiatives can be tailored to meet the diverse needs of the population.

## Limitations

This study is constrained by its narrow sample and limited geographic focus, which restricts the generalisability and transferability of the findings. The perspectives represented are predominantly from digital health experts, with limited participation from key stakeholder groups, thereby narrowing system-level insights. Consequently, the results may not reflect the variability present in low-resource or highly decentralised environments. Broader sampling across regions and professional roles in future studies would enhance external validity and better capture the diversity of healthcare contexts.

The primarily qualitative design further limits generalisability, as the absence of complementary quantitative measures precludes estimation of effect sizes or causal relationships on outcomes such as clinical decision-making, error reduction, or long-term patient trajectories. The findings should therefore be interpreted as context-specific and exploratory rather than indicative of population-level evidence. Future research would benefit from mixed-methods and longitudinal designs incorporating validated metrics to quantify impacts and assess robustness across settings.

Finally, the study does not systematically investigate mechanisms for fostering multistakeholder collaboration to support interoperability, such as policy instruments, governance structures, or incentive models, which limits its utility for informing coordinated implementation at regional or national scales. Further empirical work should evaluate collaborative models and cross-sector governance frameworks to identify actionable strategies for building cohesive and efficient digital health ecosystems.

## Study contributions

This study contributes to the growing body of knowledge on the structural and institutional factors that shape digital health outcomes, offering a nuanced understanding of how the digital divide and interoperability gaps interact to produce inequitable healthcare delivery in South Africa. By conceptualising equity as the outcome of this interplay, the study provides a framework for designing interventions that address both access and system connectivity as mutually reinforcing priorities. The findings also underscore the importance of systemic change, highlighting that incremental improvements in infrastructure or individual systems are insufficient to address the deeply rooted inequities in South Africa’s healthcare landscape. Instead, a coordinated, equity-focused approach is needed, one that integrates technological innovation with structural reforms to create a digital health ecosystem that works for everyone. Digital health technologies hold immense potential to transform healthcare delivery in South Africa, but their success depends on the deliberate and systemic dismantling of the barriers that perpetuate inequity. By addressing the digital divide, fostering interoperability, and embedding equity into every stage of digital health implementation, South Africa has the opportunity to build a more inclusive, just, and effective healthcare system for all its citizens.

## Conclusion

Across South Africa, limited digital access and weak interoperability jointly shape patterns of healthcare inequity, restricting participation in digital systems for some populations while reducing the benefits for others. Together, these constructs shape the equity of healthcare delivery, with under-resourced public facilities and marginalised populations bearing the greatest burdens. Investments in infrastructure, digital literacy, and national interoperability frameworks are critical for creating a cohesive and inclusive digital health ecosystem. Furthermore, it is imperative that equity is embedded as a guiding principle in the design and implementation of digital health systems, ensuring that benefits are distributed fairly across all populations. By addressing these challenges, South Africa can move closer to achieving equitable healthcare outcomes and unlocking the full potential of digital health for all. Without tackling the full spectrum of digital divides spanning infrastructure, skills, and outcomes, digital health risks becoming yet another mechanism that reinforces existing inequalities rather than alleviating them. Similarly, without robust interoperability, even well-resourced and connected organisations may struggle to deliver the coherent, continuous care required to improve patient outcomes equitably. Digital health technologies hold transformative potential, but their success depends on the ability to reduce, not exacerbate, inequities through deliberate, equity-centred design and implementation.

## Supplemental material

Supplemental material - Bridging digital health gaps in South Africa: A qualitative study of the digital divide, interoperability and health equity Supplemental material for Bridging digital health gaps in South Africa: A qualitative study of the digital divide, interoperability and health equity by Saidi Trust, Pavel Nenad, Gezani Parisa and Pikkarainen Annika Minna in Digital health.

## Data Availability

The datasets used and/or analyzed during the current study are available from the corresponding author upon reasonable request.*
