# Supplementary material for: Bridging digital health gaps in South Africa: A qualitative study of the digital divide, interoperability and health equity
Source: Digit Health. 2026 Apr 17;12:20552076261440967. doi: 10.1177/20552076261440967 (PMC13100368; doi:10.1177/20552076261440967)
Supplement: Supplemental material - Bridging digital health gaps in South Africa: A qualitative study of the digital divide, interoperability and health equity [file sj-pdf-1-dhj-10.1177_20552076261440967.pdf]

Appendix 1

Interview Guide

Bridging Digital Health Gaps in South Africa: The Digital Divide, Interoperability and Health Equity

The digital revolution offers unprecedented opportunities to transform healthcare delivery, particularly in Low- and Middle-Income Countries (LMICs), where accessible, affordable, and equitable healthcare is urgently needed. As the global push for universal health coverage intensifies, digital technologies are increasingly viewed as critical tools for bridging healthcare disparities. South Africa presents a compelling case study with its socio-economic challenges, stark healthcare inequities, and a history shaped by systemic divides. To address these issues, the government introduced the National eHealth Strategy (2012–2016) and the National Digital Health Strategy (2019–2024), aiming to harness digital technologies to enhance healthcare delivery, improve outcomes, and ensure sustainability. These strategies provide valuable lessons for advancing digital health equity globally. This paper examines South Africa's digital health journey, focusing on the interplay between the digital divide, interoperability, and health equity to uncover how digital technologies can reduce disparities and reach marginalized populations. By analyzing the drivers of digital transformation, barriers encountered, and the transition toward a digitalized healthcare system, this study offers actionable insights for scaling and tailoring digital health solutions in LMICs while addressing challenges of equity, accessibility, and affordability.

Research questions

1. What have been the key milestones in South Africa's digital health journey, and how have these developments shaped the current state of digital health across different regions and sectors?
2. To what extent have socio-economic challenges and historical disparities in healthcare access influenced South Africa's adoption of digital health technologies as a means to address inequities in healthcare delivery?
3. How have digital health innovations, as outlined in South Africa's national strategies, contributed to measurable improvements in healthcare delivery, patient outcomes, and system efficiencies?
4. What are the notable successes and achievements of South Africa's digital health initiatives, and how have they influenced healthcare accessibility, affordability, and equity at both regional and national levels?
5. What are the primary challenges and systemic barriers to the implementation of digital health strategies in South Africa, and what approaches have been employed to mitigate these challenges?

6. What roles do policymakers, health leaders, and other key stakeholders play in advancing the adoption, governance, and sustainability of digital health solutions in South Africa?
7. In what ways has the integration of digital health technologies impacted healthcare system efficiency, service delivery, and resource allocation within South Africa's public and private healthcare sectors?
8. What strategies and mechanisms have been implemented to ensure that digital health interventions effectively reach marginalized, underserved, and hard-to-reach populations in South Africa?
9. What lessons can be drawn from South Africa's experience with digital health transformation that could inform the design and implementation of digital health strategies in other Low- and Middle-Income Countries (LMICs)?
10. What key priorities and strategic actions are necessary to further promote digital health equity and inclusivity in South Africa, and what are the potential implications of these advancements
